# Conference Highlights of the 16^th^ International Conference on Human Retrovirology: HTLV and Related Retroviruses, 26–30 June 2013, Montreal, Canada

**DOI:** 10.1186/1742-4690-11-19

**Published:** 2014-02-24

**Authors:** Benoit Barbeau, John Hiscott, Ali Bazarbachi, Edgar Carvalho, Kathryn Jones, Fabiola Martin, Masao Matsuoka, Edward L Murphy, Lee Ratner, William M Switzer, Toshiki Watanabe

**Affiliations:** 1 Département des sciences biologiques and BioMed Research Center, Université du Québec à Montréal, Room SB-3335, 2080 St-Urbain, Montréal, Québec, Canada; 2 VGTI Florida, Port St., Lucie, FL, USA; 3 Lady Davis Insitute, Montreal, Canada; 4 Department of Internal Medicine, American University of Beirut, Beirut, Lebanon; 5 Immunology Department, Universidade Federal da Bahia (UFBA), Salvador, Bahia, Brazil; 6 Basic Research Program, Cancer and Inflammation Program, SAIC-Frederick, Inc., Frederick National Laboratory for Cancer Research, Frederick, MD, USA; 7 Centre for Immunology and Infection, Department of Biology, Hull and York Medical School, University of York, York, UK; 8 Laboratory of Virus Control, Institute for Virus Research, Kyoto University, Sakyo-ku, Kyoto, Japan; 9 University of California San Francisco and Blood Systems Research Institute, San Francisco, CA, USA; 10 Department of Medicine, Washington University School of Medicine, St. Louis, MO, USA; 11 Laboratory Branch, Division of HIV/AIDS Prevention, National Center for HIV, Hepatitis, STD, and TB Prevention, Centers for Disease Control and Prevention, Atlanta, GA, USA; 12 Graduate School of Frontier Sciences, The University of Tokyo, Tokyo, Japan

**Keywords:** HTLV-1, HTLV-2, BLV, Foamy virus, Adult T-cell leukemia/lymphoma, HTLV-1-associated myelopathy/tropical spastic paraparesis

## Abstract

The 16th International Conference on Human Retrovirology: HTLV and Related Retroviruses was held in Montreal, Québec from June 26^th^ to June 30^th^, 2013 and was therefore hosted by a Canadian city for the first time. The major topic of the meeting was human T-lymphotropic viruses (HTLVs) and was covered through distinct oral and poster presentation sessions: clinical research, animal models, immunology, molecular and cellular biology, human endogenous and emerging exogenous retroviruses and virology. In this review, highlights of the meeting are provided by different experts for each of these research areas.

## Introduction

The biannual conference on Human Retrovirology: HTLV and Related Retroviruses was hosted in Montreal (Canada) from June 26^th^ to June 30^th^ 2013. The meeting was attended by over 260 participants, arriving from all five continents. From a total of 221 submitted abstracts, 148 were selected for poster presentations, while 49 were chosen for oral presentations during regular meeting session, and 24 were selected for oral presentations during workshop sessions. To encourage attendance by promising young students, a total of twelve travel awards were provided. At the end of the meeting, the following awards were announced: the Quality Award in Basic Science was given to Jean-Michel Mesnard and Masao Matsuoka, the Quality Award in Clinical and Translational Research was given to Ali Bazarbachi and Olivier Hermine and the Quality Award for Rest of the World was received by Anna Barbara Carneiro-Proeitti. Edward Murphy received the Dale McFarlin prize and the poster presentation awards went to Chloé Journo, Anat Melamed and Megan Romeo.

The main topic of the meeting is HTLV-1 (human T-cell leukemia virus type 1), which has been estimated to infect nearly 20 million individuals worldwide [1]. This virus is associated with two important diseases: adult T-cell leukemia/lymphoma (ATLL) and HTLV-1-associated myelopathy/tropical spastic paraparesis (HAM/TSP), although other diseases have also been associated with infection by HTLV-1 [2]. In contrast, HTLV-2 has only been associated with a HAM-like disease, while newly discovered HTLV-3 and HTLV-4 viruses have yet to be associated with disease [3,4]. Non-human viruses, akin to HTLV viruses, such as STLV-1, are also being studied in simian models and can be associated with a simian form of ATLL. A very important model, the bovine leukemia virus (BLV), is known to induce B lymphoma in sheep and bovines [5]. These various viruses and their impact on human and animal health were discussed throughout the meeting, in sessions encompassing Clinical Research, Animal Models, Immunology, Epidemiology, Molecular & Cellular Biology, Human Endogenous & Emerging Exogenous Retroviruses, and Virology. As part of a series of workshops, a clinical trial group session was further included to this meeting. In this review, we present a series of summaries covering each of the sessions of the 16^th^ International Conference on Human Retrovirology.

### Clinical trial study groups

At the 16^th^International Retrovirology Conference, we were given the opportunity to attend the HAM/TSP and ATLL clinical trial workshop on the conference day out. Prior to this event we were able to hear HTLV affected patients share their plight with us at the opening ceremony and at an informal meeting.

Three issues were identified to be close to patients’ hearts:

•Patient and clinician education about HTLV and HTLV-associated diseases to improve diagnosis, access to experts in the field and reduce stigmatization

•Development of internationally recognised, evidence-based treatment protocols for HTLV-associated diseases

•Reduction or elimination of HTLV viral load

The aim of this workshop was many fold:

To take patients concerns into account and to increase the awareness of the lack of evidence based treatment particularly for HAM/TSP

•Increase awareness of the lack of clinical trials planned for HTLV-1-associated disease

•The need for more proof-of-concept clinical trials with the aim of advancing suitable drugs for next phase clinical trials

•The need to actively seek out international partners to design clinical trials with effective sample sizes

•And last but not least the need to include patients’ views and active participation in clinical trial workshops and development

We tried to reach our aims by creating an atmosphere of open discussion and debate. Scientists from both genders and many different ethnic backgrounds and HTLV endemic countries were invited to express their opinions on biomarker and diagnostic research as well as future treatment options for HTLV-associated diseases. Markers of disease stage/type differentiation, progression and treatment response were discussed for both HAM/TSP and ATLL. Over the past years, it has become very apparent that both patient groups need very individualised and tailored assessments and treatments [6-8].

The treatment of ATLL should be adapted to the clinical presentation. The combination of zidovudine (AZT) and interferon-alpha (IFN) is effective in the leukemic subtypes of ATLL and should be considered as standard of care first line therapy in that setting [9-11]. In order to prevent the occurrence of resistance and relapse after achieving complete remission, clinical trials assessing additional targeted therapies such as the combination of arsenic and IFN, Histone Desacetylase (HDAC) inhibitors or monoclonal antibodies, particularly the promising anti-CCR4 antibody, are urgently needed [6,12-14]. Currently, due to the poor outcome of patients with aggressive ATLL (acute and lymphoma forms), phase II studies are necessary in the near future. In chronic and smoldering forms, it is time to set up randomized phase III studies to assess the efficacy of the addition of new drugs for patients treated with AZT and IFN, in order to achieve disease eradication rather than long term disease control, which will allow eventual treatment cessation.

Currently, sodium valproate, Humik beta1 anti-CD122 and raltegravir are being tested in HAM/TSP patients and corticosteroid trials are planned (HAMLET-P trial, HAM/TSP Clinical Trial Study Group) [15-17]. The efficacy of AZT and IFN in ATLL warrants testing this combination in HAM/TSP patients, particularly at early stages of the disease. Some trialists would like to see anti-CCR4 antibodies tested in this cohort too. Other drug candidates are cyclosporin A and methotrexate. Eradication of HTLV might be possible by elimination of infected monocytes and through allogeneic hematopoietic stem cell transplantation [18]. CNS imaging has advanced so that asymptomatic carriers may be distinguished from patients with early HAM/TSP [19]. New imaging techniques were presented which could potentially be used to also measure and monitor treatment response.

In summary, we hope that this workshop allowed patients to be heard and facilitated collaborations between groups located on different continents by giving a human face to the diseases as well as expert groups. Through active patient participation and international collaborations, we will be able to design trials that address patients’ needs directly with meaningful sample sizes reaching representable statistical power.

### Clinical research

In the clinical research session, a total of 47 abstracts were submitted, which included 18 abstracts on ATLL, 25 on HAM/TSP, 4 on related diseases, and one on co-infection. The distribution of countries from which abstracts are coming, despite a certain bias for ATLL studies from Japan, highlight the strong international interest that these diseases has led to and the shared efforts by a multitude of international research teams in achieving viable alternative treatments. The international distribution of abstracts also indicates that it is more difficult to collect a large numbers of ATLL patients in many countries beside Japan, whereas it is relatively feasible for HAM/TSP patients, although the funding capacity of a given country is an important factor, which can impact on the ease of patient recruitment.

Abstracts on ATLL are categorized into clinical trials, pathophysiology and diagnosis. As for the treatment of ATLL, reports on chemotherapy, antiviral therapy and antibody therapy were alternatively presented. Utsunomiya reported the results of a phase II study of LSG15 (chemotherapy) with mogamulizumab, an anti-CCR4 monoclonal antibody [20]. The results showed higher complete remission (CR) rate in the group treated with mogamulizumab than the control group (no mogamulizumab), suggesting promising effects of combined therapy. Maeda *et al.* presented results of a mogamulizumab monotherapy on chemotherapy-resistant patients [21]. All 8 patients showed CR, which is striking and will need to be carefully evaluated. Yonekura *et al.* reported high rate of CR with skin lesions in 4 out of 6 patients treated with mogamulizumab monotherapy [22]. These data underline the importance of additional clinical studies evaluating the usefulness and the modality of mogamulizumab. However, none of the studies compared this new treatment with basic standard therapy proposed in other countries, namely interferon plus zidovudine as a first line treatment for treatment of naïve acute ATLL patients and in combination with chemotherapy in the treatment of naïve lymphomatous ATLL. Such comparison would more realistically reflect the survival benefit of mogamulizumab in patients with ATLL.

With respect to current antiretroviral therapy (AZT/IFN), four abstracts described results of this treatment. Among them, Pimentel *et al.* reported a retrospective analysis of their results of 89 patients, who were either treated with chemotherapy, AZT/IFN or both [23]. While their results did not provide new information, they are slightly different from those reported previously, comparing results between chemotherapy and AZT/IFN therapy in a single institution. Hodson *et al.* presented results depicting molecular analysis of four patients with chronic type ATLL after AZT/IFN therapy [24]. They suggested the usefulness of new molecular techniques for proviral load and clonality analyses (currently used as a standard in clinical care in some countries), which actually led to the identification of one cured patient with the disappearance of a dominant clone. Kchour *et al.* rather focussed on cytokine analysis of patients treated with AZT/IFN combined with arsenic and argued that this therapy induced restoration of an “immunocompetent-like” microenvironment, although they did not directly analyze stromal cells [25]. However, patients did display a high response rate and CR rate as well as a shift of a cytokine expression profile from a Th2 to a Th1 response.

Regarding other therapeutic modalities, Belrose *et al.* focussed on the effect of valproate (VPA) on Tax, Gag and HBZ expression in *ex vivo* cultured ATL cells [26]. Interestingly, VPA suppressed HBZ expression and increased *tax* and *gag* mRNA levels. Oka *et al*. showed the potential utility of photodynamic therapy [27]. Phillips *et al.* reported results of a phase II study of lenalidomide in patients with relapsed or refractory ATLL [28]. In this small study, lenalidomide showed limited clinical activity and manageable toxicity, as reported last year by a Japanese group.

Suehiro *et al.* have highlighted results of a phase-I study of a therapeutic vaccine [29]. Patients were vaccinated by autologous dendritic cells pulsed with peptides corresponding to Tax-specific CTL epitopes. So far, two patients were enrolled in the study and their clinical outcomes were partial remission and stable disease. Impressively, these results demonstrated that solely targeting Tax in ATLL suffices to induce a significant response.

Takemoto *et al.* showed the potential diagnostic utility of serum sCD25 and sCD30 levels [30]. By studying 60 patients, Pornkuna *et al.* further suggested that sCD30 could be a new serum biomarker to predict two-year overall survival of ATLL patients [31]. On the other hand, Amano *et al.* presented results of a pilot study on the immunotherapeutic potential of varicella vaccine in smoldering and cutaneous ATLL [32]. Results appeared promising since the median overall survival was 24 months, which was significantly longer than previously reported findings (16 months). Kagdi *et al.* demonstrated that, through multi-color FACS analysis combined with integration site (IS) analysis, CD127 expression was an important and significant diagnostic tool [33].

As for HAM/TSP, there were many reports dealing with HAM/TSP treatment, although they included a limited number of patients and need further studies. Such examples are listed: safety and efficacy of a humanized monoclonal anti-IL15Rβ antibody (CD122) (Massoud *et al.*) [34], potential use of BNZ-gamma peptide (selectively blocking binding and downstream signaling of IL-2, IL-9 and IL-15) as a new drug (Massoud *et al.*) [35], clinical study using Infliximab (anti-TNF-α monoclonal antibody) (Martin *et al*.) [36], efficacy of Methotrexate (Ahmed *et al*.) [37] and efficacy of Fampridine (a selective neuronal potassium channel blocker) (Menna-Barreto) [38]. One of the problems in these reports is the lack of standard criteria for treatment efficacy, which makes it difficult to compare the results from the different clinical research teams. From this point of view, it is important that reports focus on this problem. As such, Adonis *et al.* reported the usefulness of the 6 minute walk and 10 m timed walk (10mTW) [39]. The latter is also evaluated as a primary outcome measure of treatment response in a multicenter international collaborative study, HAMLET-P (Martin *et al*.) [40].

CXCL10 and neopterin in cerebral spinal fluid (CSF) were reported as candidate prognostic biomarkers for the evaluation of clinical status and of drug effects (Yamano *et al.*) [41]. The results were cross-validated in two patient groups. Spinal cord cross-sectional area measured by MRI was also presented as another biomarker candidate. It is expected that these candidate markers will be evaluated by international multi-institutional collaborative prospective studies to increase the evidence levels.

As for the clinical epidemiology of HAM/TSP, poor prognostic factors have been reported by cohort studies. Examples include levels of provirus DNA copies and gender (female). Factors that affect the health and the quality of life in patients were highlighted at this meeting, such as HIV co-infection and physical inactivity. Nation-wide epidemiological studies with a novel patient registration system in Japan appears to be an important improvement (Coler-Reilly *et al.*), which is expected to evolve into an international system because of the relatively small number of HAM/TSP patients [42]. Three abstracts described familial clustering of HAM/TSP patients (Nozuma *et al.*, Coler-Reilly *et al.*, and Alvarez *et al*.) [42-44]. Results from Nozuma *et al.* and Coler-Reilly *et al.* suggested positive clustering and lower age of onset [42,43]. On the other hand, Alvarez *et al.* did not find clustering when comparing HAM/TSP patients to asymptomatic carriers, although there are some families where HAM/TSP patients show clustering [44]. These sets of information appear to be important for evaluating genetic risks for HAM/TSP development.

Studies on clinical features of HAM/TSP were further presented in three abstracts. A high incidence of pulmonary lesions was reported by Honarbakhsh and Taylor. Boa-Sorte *et al*. noted an association between HAM/TSP and depression, while Gascón *et al.* provided evidence for a possible relationship between the neurocognitive performance and the degree of depression [45-47]. Tanajura *et al.* and Matsuzaki *et al.* demonstrated that follow-up of asymptomatic carriers is useful for early diagnosis of HAM/TSP [48,49]. Risks for HAM/TSP development were further described in two abstracts. Okajima *et al.* reported an association of skin lesions with the high risk for HAM/TSP and De Lourdes Bastos *et al.* showed a higher frequency of HAM/TSP for carriers with tuberculosis [50,51].

Labanca *et al.* have shown that dizziness without an apparent cause in HTLV-1-asymptomatic carriers may be a useful symptom for early diagnosis of HAM/TSP [52]. In this respect, galvanic vestibular stimulation may thus be a useful test for diagnosis (Cunha *et al.*) [53]. A high provirus load is a well-known risk factor for HAM/TSP. Digital droplet PCR is a new promising method to measure the copy number of provirus DNA and was reported by Brunetto *et al.*[54]. Furthermore, Bassi *et al.* claimed the need for an international, cross-sectional study with standardized methodologies for measuring the copy numbers of provirus DNA [55]. International collaboration is expected to establish a standard method and criteria for evaluating the risks.

Concerns regarding the HAM/TSP diagnostic criteria proposed by WHO in 1989 were underscored by Bassi *et al.*[55]. For improving diagnosis, MRI studies of the brain and the spinal cord was evaluated. However, it was shown that these approaches did not currently have enough sensitivity or specificity (Bastos *et al.* and Romanelli *et al.*) [56,57]. Presence of immune activation and immune response to the virus in asymptomatic carriers may be the cause for the difficulties in identifying diagnostic markers (Bahia *et al.* and Matsuura *et al.*) [58,59].

Another disorder associated with HTLV-1 infection was presented by Einsiedel *et al.*, who reported an association with bronchiectasis in Indigenous Australians [60]. Bronchiectasis patients showed a higher provirus load and higher risks for shorter life expectancy. Detailed studies appear to be required to describe health problems in Indigenous Australians with HTLV infection.

### Animal models

The current overview summarizes animal models for HTLV-1 and related viruses, and highlights a few key studies and presentations. An excellent recent review by Bazarbachi and his colleagues provides an overview of animal models for delta-retroviruses which include HTLV-1, 2, BLV, and STLV-1 [61]. The studies have been performed in non-human primates, rabbits, rats, and mice.

A recent review by Willems and colleagues highlights the similarities of the genomes of HTLV-1 and BLV, and points out that BLV has a worldwide distribution although it has been effectively eradicated from Europe [62]. One third of infected cattle develop lymphocytosis, and 3-5% develop leukemia after 4–10 years. Presentations by Rodriguez *et al.* and Martinez *et al.* described the use of the BLV model for development of vaccines [63,64]. In particular, Rodriguez *et al.* described an attenuated vaccine that efficiently and persistently protects against BLV in real herd settings [64].

Another interesting application of this model was the demonstration in 2005 that the histone deacetylase inhibitor, valproate, activates BLV expression and triggers apoptosis, and induces leukemia regression *in vivo*[65]. The authors hypothesized that this was due to immunological clearance of cells expressing previously hidden BLV antigens. This finding led to a clinical trial in HAM patients, resulting in transient increases in proviral load, which subsequently declined to levels below the baseline level [66]. A study by Mahieux and colleagues in STLV-1 experimentally infected baboons, showed that the combination of valproate and azidothymidine resulted in a strong decline in proviral load [12]. Altogether, these studies are important in opening the door to investigations of HTLV latency, methods to reactivate and eradicate latently infected cells, and possible clinical benefits of depressing proviral load.

The rabbit model of HTLV-1 transmission is described in an excellent recent review by DucDodon and Lairmore [67]. These inbred, although not genetically identical rabbits, can be infected with HTLV-1-infected rabbit or human cells, and examined over the next 8 wks or longer for anti-HTLV-1 humoral responses, and cells in different lymphoid compartments for proviral load, and *ex vivo* culture studies of viral structural and regulatory protein expression. This model, which resembles asymptomatic infected humans, has been useful in defining key molecular determinants involved in virus replication, utilizing the infectious molecular clone, ACH, that Jason Kimata constructed and characterized in Ratner’s lab almost 20 yrs ago [68-72].

In a comprehensive study published by Franchini and colleagues, HTLV-1 variants with mutations in p12, p30, or HBZ were examined in human dendritic cells (DCs) in culture, as well as in rabbits and rhesus macaques [73]. They found that both p12 and p30, but not HBZ, were important for virus production from human DCs. However, in rabbits, the p12 and p30 knockout (KO) viruses showed a significant decline in virus levels only at week 16, whereas lower levels of virus were found with HBZ KO viruses at all time points. However, very different results were found in macaques, where animals exposed to the p12 KO virus failed to seroconvert, and only 1 of 3 animals exposed to the p30 KO virus fully seroconverted. All 4 animals exposed to HBZ KO viruses seroconverted. Thus, the seroconversion data in macaques correlated with the data on virus infection of human DCs in culture. However, reversion of HBZ mutations were found in all macaques studied, suggesting an important role of HBZ in macaques. Additional studies of STLV-1 in non-human primates were described in the abstracts by Miura *et al.*, Pise-Masison *et al*. and Souquiere and Kazanji [74-76].

There have been numerous HTLV-1 transgenic animal models, most of which have focused on Tax, as summarized in a recent review by Ohsugi [77]. These mouse models have utilized various promoters to drive Tax expression, including the viral, CD4, and metallothionein promoters that resulted in arthritis and other inflammatory disorders. Notably, models with the granzyme B promoter, lck proximal or distal promoters have resulted in leukemia/lymphoma-like diseases. It is important to recognize that none of these models gave rise to a disease similar to HAM, and the single example cited in this review (and Ohsugi *et al*.) [78] of transgenic mice with paraparesis was due to lymphomatous involvement of the spinal cord, which is quite distinct from the pathophysiology of HAM.

In the GzmB-Tax model utilized in the Ratner laboratory, 100% of the animals developed tumors on the tails, ears, extremities, with involvement of the spleen, blood, bone marrow, osteolytic bone metastases and hypercalcemia, resembling the peripheral skin disease, leukemia and bone disease developing in patients with ATLL [79,80]. However, the majority of these murine tumors were NK-T precursor cell tumors. Use of this model was facilitated by studying bioluminescence with an indicator to non-invasively examine Tax expression based on the firefly luciferase (LUC) gene driven under the regulation of the viral LTR [81]. These double transgenic TAX-LUC animals showed dynamic Tax activity and activation of NF-κB. Moreover, granulocyte infiltration was visualized by conversion of luminol to a bioluminescent emitter [82]. These studies provide a highly sensitive tool to detect neoplastic lesions in as few as 50 cells. One example of the use of this animal model to investigate the NF-κB target gene, IL-15, was described in the oral presentation by Rauch *et al*. [83].

An intriguing transgenic mouse model with Tet-inducible Tax was developed by Greene and colleagues [84]. These mice developed a T cell inflammatory skin disease. When Gzm-rTA mice were bred with the transgenic mice expressing Tax under the regulation of the Tet-responsive promoter, these mice developed a Tax-dependent CD4+ tumor that involved lymph nodes, liver, lung, and spleen, and regressed upon withdrawal of Tax expression (Rauch & Ratner, unpublished).

One particularly interesting application of proximal Lck - Tax transgenic mice has been to identify cancer stem cells from these tumors, representing a rare CD117+ cell type [85]. This was followed by an interesting study of Bazarbachi and colleagues, using cells from these transgenic animals [86]. They demonstrated that the combination of arsenic and interferon did not affect the growth of tumors in treated mice, but instead blocked the transplantability of these tumors into secondary recipients. Tumor cells from As/IFN-treated mice exhibited markedly enhanced levels of apoptosis in secondary recipients as compared to untreated animals. This work provides an important clue to understanding the biology, chemoresistance, and possibly an effective therapy of ATLL directed at cancer stem cells. A study of an HSP90 inhibitor using this model was presented by Ikebe *et al*. [87].

A few transgenic animal models have been constructed with other HTLV genes. Of note, transgenic animals expressing HBZ under the regulation of the murine CD4 promoter, reported by Matsuoka and colleagues, all developed inflammation, and about 40% of animals at 2 yrs of age or older exhibited a CD4+ T cell lymphoma [88]. Ratner and colleagues found that transgenic animals expressing HBZ under the regulation of the granzyme B promoter developed a non-lethal T cell malignancy with 100% incidence (Rauch & Ratner, unpublished). Moreover, their preliminary data suggests accelerated tumor development in transgenic animals expressing Tax and HBZ compared to those expressing Tax alone.

A particularly exciting approach utilized to model many different viral infections has been the use of humanized mice. In the review by DucDodon, the generation of humanized mice from sublethally irradiated newborn Rag2−/− gammac−/− animals by intrahepatic injection of cord blood CD34+ cells is described [67]. By 6 wks of age, human cells are found in the bone marrow, thymus, spleen, and peripheral blood of these mice.

Intraperitoneal injection of irradiated HTLV-1-immortalized MT2 cells in these animals resulted in infection, and a high proviral load in a subset of these animals, 20–40 wks after transplantation [89]. Animals with high proviral load manifested HTLV-1-positive cells in the thymus and spleen, expansion of single CD4+ and CD8+ cells with CD25 expression, NF-κB activation, as well as lymphoma with clonal integration of the HTLV-1 provirus. This is an important model that has the potential to explore questions about pathogenic determinants of HTLV-1 and interactions with human cellular determinants in an ATLL animal model, as well as applications to investigations of novel therapies or vaccines, as described in abstracts by Saito *et al*. and Tezuka *et al*. [90-92].

A major shortcoming of animal models has been the failure to develop an appropriate animal model for HAM. Nevertheless, there are many new opportunities to use animal models to explore questions about the determinants of virus transmission, pathogenesis, latency, retroviral replication, clonal proliferation of infected cells, virus spread, and ATLL stem cells, in order to develop methods to prevent infection, develop vaccines, effective therapies, and understand the role of stem cell transplantation in the treatment of ATLL.

### Epidemiology

After 30 years of HTLV epidemiology research, much has been learned, but there are still gaps in our knowledge about this human retrovirus. HTLV-1 and −2 prevalence is well described in many countries; transmission is well understood; pathogenesis research has yielded much knowledge about disease mechanisms of ATLL and HAM/TSP; and the clinical manifestations of ATLL and HAM/TSP have been well described. However, there are significant knowledge gaps regarding prevalence (Africa & Asia) and secular trends are largely unstudied; the effectiveness of prevention strategies is poorly described; prognosis at single-patient levels is still elusive; and patient-centered research on symptoms is in its infancy: what does “living with HTLV” mean?

During the oral session of the meeting, Gessain and Cassar presented their recently published estimate of 5–10 million HTLV-1 carriers in the world [93,94], which is lower than the previous estimate of 10–20 million [1]. Perhaps, more important than a single number, this work identified gaps in HTLV-1 prevalence data, especially in East and North Africa, China and India. It was also agreed that a similar review of literature and global estimate would be very useful for HTLV-2 infection.

Usadi *et al.* presented data on telomere length in *ex vivo* peripheral blood mononuclear cells (PBMCs) from asymptomatic HTLV-1- and HTLV-2-infected subjects [95]. Overall, there was no difference in telomere length between infected and uninfected individuals after matching for age and other demographic characteristics. Interestingly, HTLV-1-infected subjects did not show an age-related decline in telomere length. Finally, among HTLV-2-infected persons, there appeared to be an association between shorter telomere length and the abnormalities in vibration sense.

Pataccini *et al.* presented a very detailed comparison of enzyme immunoassay (EIA) test performance in the setting of blood bank screening in Latin America [96]. A total of 14 HTLV-1, 13 HTLV-2 and 233 negative samples were tested with five different EIAs. All tests had 100% sensitivity, but there was substantial variation in specificity ranging from 93.1% to 99.1%. This information will be valuable to blood bankers when they choose an EIA assay to minimize false positive reactivity, which can result in the loss of blood units and potential false notification of blood donors regarding HTLV infection.

De Campos *et al.* presented research on the impact of urinary incontinence on the quality of life of a cohort of HTLV-1-infected women in Salvador, Brazil with overrepresentation of HAM cases [97]. Bladder dysfunction was very common, with a 61% prevalence of urinary incontinence, and a 71% prevalence of detrusor hyperactivity, and 29% with sphincter dyssynergy. Of particular interest, data showed a substantial adverse impact of bladder symptoms on the quality of life among these women.

Finally, dos Santos *et al.* presented work indicating that the IL28B gene polymorphism SNP rs8099917 allele GG is associated with HTLV‒1‒associated myelopathy/tropical spastic paraparesis (HAM/TSP) [98]. Her work, analogous to a similar IL28 gene polymorphism associated with reduced clearance of hepatitis C virus, suggests that host genetic variation may be associated with poor control of HTLV-1 infection and thereby increase the risk of neurologic disease.

There were a total of 15 posters presented at the conference; highlights are noted below, by geographic region.

Brazil: Gadelha *et al*. analyzed a large cross-sectional study of 2,766 pregnant women in the southern Bahia, Brazil and found a confirmed HTLV-1 prevalence of 1.05% [99]. The same group reported genetic similarities between HTLV-1 viral isolates obtained from Brazil and those obtained from Mozambique and South Africa, suggesting that African slaves imported into Brazil may have originated from or transited through Southern Africa before transportation to Brazil [100].

The GIPH cohort from Brazil reported that, among 233 HTLV-1-positive individuals examined by a neurologist, 12% were diagnosed with HAM/TSP and there was a high prevalence of other symptoms including myalgia, urinary incontinence and gait abnormalities [101]. This prevalence should not be confused with HAM/TSP incidence, which the same group has previously published as 5.3 cases per 1,000 HTLV-1-seropositive cases per year (95% confidence interval: 2.6 -10.9) [102]. The same group charted family trees to infer vertical versus sexual transmission in 95 family groups [103]. In 23 (24%) of the families, transmission occurred vertically and 58 (61%) occurred via sexual transmission, highlighting the importance of sexual transmission in maintaining HTLV-1 prevalence in an endemic population.

USA: Chang *et al*. reported on the prevalence of HTLV among 2 million first-time, United States blood donors during 2000 – 2009 [104]. A total of 104 (5 per 10^5^) had HTLV-1 antibodies and 300 (14.7 per 10^5^) had HTLV-2 antibodies. HTLV-1 was associated with female sex, older age, and black and Asian race/ethnicity, while HTLV-2 was associated with female sex, older age, non-white race/ethnicity, lower education and residence in the Western and Southwestern United States. Overall, prevalence had declined since the 1990s, and there was evidence of a decreasing birth cohort effect. Finally, Switzer and colleagues reported a cross-sectional study of 234 US thalassemia patient sera collected in 2008, of whom 3 (1.3%) were confirmed HTLV-seropositive [105,106]. These data support HTLV testing of other patients requiring further transfusions.

United Kingdom: Croxford *et al*. reported baseline data on 892 cases of HTLV-1 in England and Wales from 2003 to 2012 from the UK National Reference Laboratory; the cohort will be followed prospectively [107].

Australia: Cassar *et al.* reported on the molecular epidemiology of HTLV-1 in Australia, finding that the Australian isolates belonged to HTLV-1 subtype C (the Melanesian subtype), and that two variants mapped separately to the north versus south and central regions of Australia [108].

Africa: Filippone *et al*. reported additional data on potential interspecies transmission of HTLV-I in 269 Bantu and Pygmy subjects from rural Cameroon, who had been bitten or otherwise exposed to primates [109]. HTLV-1 prevalence was 23/269 (8.5%) among bitten individuals versus 4/269 (1.5%) in controls without primate bites. Fox *et al*. reported on HTLV-1 seroprevalence among 418 paired mother and child samples from healthy mothers and children at a hospital in Malawi [110]. Three (0.72%) women were HTLV-1-positive and 8 (1.9%) women had HTLV-2 infection; of these 11 women, two children were also seropositive, indicating probable mother to child transmission at rates consistent with those reported in literature.

BLV: Choudhury *et al*. reported an interesting story of false positive BLV tests due to a colostrum replacer with passive acquisition of anti-BLV antibodies in cattle without BLV infection [111].

The author suggests a few important priorities in HTLV epidemiology research. First, we need to fill in gaps in HTLV-1 prevalence in Africa, China and India; second, monitor secular (time) trends in well-defined populations (e.g. blood donors and pregnant women); third, search for new human retroviruses in concert with other viral discovery efforts in humans and animals; finally, larger molecular epidemiology studies of selected populations of interest (Caribbean, Bakola pygmies, Amerindians) are needed to study puzzles on historical origins of HTLV-1 and −2. Prevention efforts to limit HTLV infection, including bottle feeding by HTLV-infected mothers and condom usage, need to be implemented in HTLV endemic populations. We need better epidemiological monitoring of the effectiveness of such targeted (and coincidental, HIV- or STI-related) prevention interventions. Finally, the association between transmission route and disease outcomes needs to be better defined and changes in disease incidence in response to shifts in transmission routes need to be monitored.

### Immunology

HTLV-1 infects CD4+ CD25+ T cells with a contribution to transmission by cells of the myeloid lineage. The immunopathogenic consequences of HTLV-1 infection are profound and reflect one of the most active areas of HTLV-1 research. The International Conference on Human Retrovirology in Montreal reflected this high level of activity with many important contributions regarding immunopathogenesis and immunotherapy of HTLV-1 infection.

How human retroviruses are initially recognized by the innate immune system and how that sensing of infection is transmitted to generate a robust immune response has been a poorly understood area of viral immunology. Recent advances in the identification of sensors that recognize incoming pathogens have begun to reveal the mechanisms underlying the early host response to HTLV-1 infection. Sze *et al.* investigated the mechanisms underlying myeloid cell infection by HTLV-1 and demonstrated that HTLV-1 infection induced apoptosis of monocytes in a manner dependent on SAMHD1, a deoxynucleoside triphosphate triphosphohydrolase that functions as a restriction factor to limit HIV-1 replication [112]. A 90 nucleotide replicative intermediate from the U5 region of HTLV-1 bound to the DNA sensor STING, and mediated the antiviral response via IRF3 activation. These authors further demonstrated that STING-mediated apoptosis in infected monocytes required the generation of a pro-apoptotic complex between IRF3 and the Bcl-2 protein Bax. In a related study, Alais *et al.* examined the involvement of dendritic cell subsets as potential viral reservoirs that spread HTLV-1 to surrounding lymphocytes [113]. Distinct monocyte-derived DC (MDDC) subsets were isolated and infected with HTLV-1 biofilm; MDDC subsets were not equally susceptible to HTLV-1 infection, with DC maturation altering susceptibility to HTLV-1 infection, suggesting that differential susceptibility of various DC subsets to HTLV-1 infection may differently shape immune responses and therefore affect viral pathogenesis.

De Castro-Amarante and colleagues analyzed genomic DNA isolated from sorted CD4+, CD8+ CD14^++^CD16^–^, CD14^+^CD16^++^, and CD14^++^CD16^+^ cells by nested PCR; in HTLV-1 patients with high proviral load (PVL), all monocyte subsets as well as CD4+ and CD8+ cells were positive for HTLV-1 [114]. In contrast, the intermediate monocytes were negative or very weakly positive for HTLV-1 in patients with low PVL. To test whether natural STLV-1 infection recapitulates infectivity by HTLV-1, monocyte subset distribution in 8 STLV-1-infected Rhesus macaques and 16 naïve animals was examined. Consistent with human infection, the frequency of intermediate monocytes was higher in infected macaques compared to naïve animals with a positive correlation between PVL and intermediate monocyte frequency.

To explore the host-pathogen interaction between DCs and cell-free HTLV-1, Rahman *et al.* evaluated FLT3 ligand-cultured mouse bone marrow-derived DCs (FL-DCs) and chimeric HTLV-1 for various immune markers [115]. FL-DCs upregulated expression of surface markers (CD80, CD86, and MHC class II) on infection; however, the level of MHC class I remained unchanged. Multiplex cytokine profiling revealed production of an array of proinflammatory cytokines and type 1 IFN (IFN-α) by FL-DCs treated with virus. Gene expression studies using type 1 IFN-specific and DC-specific arrays revealed upregulation of IFN-stimulated genes, most cytokines, and transcription factors, but a distinct downregulation of many chemokines. Another study from the same group explored the role of DCs during early HTLV-1 infection *in vivo*. A chimeric HTLV-1 with a replaced envelope gene from Moloney murine leukemia virus was used to allow HTLV-1 to fuse with murine cells. In addition, a CD11c-diphtheria toxin receptor transgenic mouse model system was used to generate conditional transient depletion of CD11c(+) DCs [116]. Infection of transgenic mice with HTLV-1 was achieved using both cell-free and cell-associated infection routes in the absence and presence of DCs. The ablation of DCs led to an enhanced susceptibility to infection with cell-free, but not cell-associated HTLV-1 in both CD4 and non-CD4 fractions. Infection with cell-free virus in the absence of DCs also led to increased levels of Tax mRNA in the non-CD4 fraction. Moreover, depletion of DCs significantly dampened the cellular immune response against both cell-free and cell-associated virus.

Therapy for HTLV-1 infection remains a challenge. As pathology in HTLV-1 infection is in part related to lymphocyte proliferation and activation, immunotherapy has been considered a potential treatment for HTLV-1. It is well known that the association of IFN-α plus zidovudine has prolonged the lifetime of patients with ATLL. A study showed how IFN-α downregulated HTLV-1 expression and also induced apoptosis of ATL transformed cells [117]. Both IFN-α and IFN-β increased apoptosis, had antiproliferative and antiviral effects, and decreased pro-inflammatory cytokine levels. AZT combined with IFN-α induced cell apoptosis in IL-2-dependent HTLV-1-infected T-cells, associated with phosphorylation of p53 and enhanced expression of genes responsive to p53. In a study by Khouri *et al*., IFN-β treatment was however significantly more effective in inhibiting viral p19 protein level and lymphoproliferation, when compared to IFN-α [118]. Van Weyenbergh described anti-CD3 antibody therapy as a treatment for transplant rejection and several autoimmune diseases; its potential in HAM/TSP had not been investigated [119]. In contrast to normal donors and patients in early disease stages, anti-CD3 treatment did not increase lymphoproliferation in PBMCs from advanced HAM/TSP patients, but strongly induced apoptosis. In addition, anti-CD3 treatment did not induce a pro-inflammatory cytokine storm, either at the protein or mRNA level. Using microarray analysis, treatment of HAM/TSP PBMCs with anti-CD3 mAb had a pronounced effect on gene expression, significantly down-regulating certain pro-inflammatory genes and up-regulating cell cycle-related and immunoregulatory genes, such as CTLA4. Ramos’ group presented data using brentuximab vedotin (SGN-35), an anti-CD30 monoclonal antibody conjugated to a potent microtubule poisoning agent monomethyl auristatin E that is effective in the treatment of CD30-expressing lymphomas [120]. The proportion of CD30+ ATLLs was 36% (95% CI 11%-61%), including 47% in lymphomatous-type, 28% in acute-type, and 10% in indeterminate cases. Four of 12 (33%) acute-type ATLL cytospin cases were CD30+; however, a high expression of 80% was observed in only one case. One patient with CD30+ acute-type ATLL with diffuse skin involvement treated with brentuximab had an objective transient response. Uto and colleagues studied the induction of cytotoxic T lymphocytes as a strategy for elimination of infected cells [121]. The immunization with PIC nanoparticles carrying HTLV-1 Tax peptide induced expansion of Tax-specific CD8^+^ T cells. In contrast, no such induction was observed with the peptide alone or peptide plus an aluminum adjuvant.

Enose-Akahata *et al.* evaluated the immune response against HBZ in HTLV-I-infected individuals [122]. The immunoreactivity for HBZ was detected in subsets of all HTLV-I-infected individuals, but did not discriminate between asymptomatic carrier, ATLL and HAM/TSP. However, the frequency of detection of HBZ-specific antibodies in the serum of ATLL patients with the chronic subtype was higher than in ATLL patients with the lymphomatous subtype. Antibody responses against HBZ did not correlate with proviral load and HBZ mRNA expression in HAM/TSP patients, but the presence of HBZ-specific response was associated with reduced CD4^+^ T cell activation in HAM/TSP patients. Rowan *et al.* searched for the role of host cytotoxic T-lymphocyte (CTL) responses in limiting expansion of HTLV-1-infected CD4 + T-cells *in vivo* and assayed the ability of equally efficient Tax-and HBZ-specific CTL clones to kill unstimulated, naturally infected cells from HLA-A*02 + HTLV-1+ individuals [123]. Infected cells, which expressed Tax during the course of the assay, upregulated surface expression of HLA-A*02, and were eliminated efficiently by Tax-specific CTL. HBZ-specific CTL killed Tax+ cells less efficiently, preferentially killing cells with high levels of HLA-A*02. Niederer *et al.* performed integration site analysis in Japanese HTLV-infected asymptomatic carriers and HAM/TSP patients to test the hypothesis that a strong CD8^+^ T-cell response to HBZ alters the frequency distribution of infected T-cell clones and selects the genomic environment of the proviral integration site (IS) *in vivo*[124]. A high-throughput protocol was used to map and quantify IS in 95 HAM/TSP patients and 68 asymptomatic carriers (ACs) from Kagoshima, Japan, and 75 ACs from Kumamoto, Japan. Individuals with 2 or more HLA class I alleles predicted to bind HBZ were classified as ‘strong’ HBZ binders. The results suggest that the predicted strength of HBZ binding does not influence the overall clone frequency distribution. However, clonal abundance was correlated with frequency of proviral integration within transcriptionally active areas in weak HBZ binders, but not strong HBZ binders.

Recent studies have shown that a large percentage of HTLV-1-infected subjects, who do not fulfill the criteria for HAM/TSP, have overactive bladder as well as other neurologic manifestations. The fact that patients with overactive bladder have an exaggerated immunologic response similar to HAM/TSP, with high production of TNF-α and IFN-γ as well as higher proviral load than HTLV-1 carriers, provides evidence that overactive bladder is an oligosymptomatic form of HTLV-1 (Santos *et al*.) [125]. As patients with HAM/TSP have already a fibrotic spinal cord and a low possibility of restoring the neurologic damage caused by viral infection, identification of other neurologic diseases that precede or are oligosymptomatic forms of HAM/TSP is a tool to evaluate efficacy of drugs against HAM/TSP.

Ciliao-Alves *et al.* investigated the expression of the human leukocyte antigen-G (HLA-G) for its immunosuppressive effects [126]. A correlation between HLA-G polymorphisms in symptomatic and asymptomatic HTLV-1-infected individuals indicated that HTLV-1-related symptoms in HAM/TSP group could be partially determined by higher expression of HLA-G. Higher expression of HLA-G may protect HTLV-1-infected cells against immune system attack, leading to the increased proviral load and HAM/TSP symptoms. Olavarria and colleagues studied HLA-A, -B and –C polymorphisms and determined the individual ancestry proportion of European, African and Amerindian in 209 HTLV-1-infected individuals in order to identify genetic factors that associate with HAM/TSP [127]. When considering only the HAM/TSP subsample, the results suggested that European ancestry were predisposed to higher PVL, while African ancestry was associated with lower PVL.

Pinto *et al.* compared the global gene expression profile of circulating CD4^+^ T cells in healthy control (CT), asymptomatic HTLV-1 carrier (HAC) and HAM/TSP group [128]. Twenty five differentially expressed genes in common between CT vs. HAM/TSP and HAM/TSP vs. HAC were identified in the granzyme A (GZMA) signaling pathways. GZMA and PRF1 gene expression were significantly increased in HAM/TSP group compared to CT and HAC groups. Foxp3 gene expression was significantly increased in HAM/TSP group. GZMA, GZMB, and PRF1 genes positively correlated with Foxp3 gene expression. Menezes *et al.* tested Fas expression and function in lymphocyte activation, apoptosis, lymphoproliferation and gene expression profiling, using flow cytometry and microarray analysis in PBMCs from HAM/TSP patients, asymptomatic HTLV-1-infected individuals and healthy controls [129]. Fas expression was increased in both asymptomatic HTLV-1-infected individuals and HAM/TSP patients, as compared to uninfected controls. In HAM/TSP, Fas expression correlated positively to lymphocyte activation markers (HLA-DR, CD86), but negatively to disease duration. Likewise, increased Fas expression in HAM/TSP did not lead to increased apoptosis upon *in vitro* culture. However, in HAM/TSP patients, IFN-α-induced Fas expression paralleled decreased lymphoproliferation.

HTLV-1 is primarily found in the CD4 + CD25+ T cell subset (Tregs), the cells that are responsible for peripheral immune tolerance and which are known to be dysfunctional in HAM/TSP. However, due to the inherent inflammatory component of HAM/TSP, markers normally used to characterize T regs, such as CD25, FoxP3, and CTLA4 are problematic in differentiating Tregs. Recent evidence has shown that FoxP3 expression and function is determined epigenetically, specifically through DNA methylation in the Treg-specific methylation region (TSDR). Anderson analyzed the methylation status of specific CpGs in the TSDR in PBMCs, CD4+ T cells, and CD4 + CD25+ T cells from normal healthy donors (NDs) and HAM/TSP patients [130]. Decreased demethylation in PBMCs and CD4 + CD25+ T cells from HAM/TSP patients as compared to NDs was demonstrated despite the increased CD4 + CD25+ frequency in HAM/TSP. Further, decreased TSDR demethylation correlated with decreased functional suppression in Treg cells of HAM/TSP patients.

Similar to other infectious diseases, a large number of HTLV-1-infected individuals remain carriers, suggesting that effective immunologic responses able to control viral proliferation can be developed. CD8^+^ T cells play a pivotal role in both protection and pathology associated to HTLV-1. It is known that the killing immunoglobulin-like receptor (KIR) genotype influences CTL efficiency by affecting HLA class I-mediated HTLV-1 immunity. The observation that, after PBMC stimulation with TAX peptides, the frequency of KIR2 DL2^+^ CD8^+^ T cells is higher in HTLV-1 carriers than in patients with HAM/TSP suggests a potential role of these cells in the control of the virus (Twigger *et al.*) [131]. HTLV-1-infected T cells can also be killed by ADCC with a rat monoclonal antibody against HTLV-1 envelope GP46 or a human polyclonal IgG purified from serum of HAM/TSP patients (Tanaka *et al*.) [132]. A vaccine able to induce or boost anti-GP46 antibody responses may have a potential for protection and therapy against HTLV-1.

Despite advances in the knowledge on the immunopathogenesis of HTLV-1 infection, the mechanisms of cellular migration to the central nervous system (CNS) and the maintenance of the inflammatory process in patients with HAM/TSP is not completely understood. HAM/TSP patients have high levels of CXCL10 in the CSF as well as a large number of cells in the CSF expressing CXCL10-binding receptor CXCR3. As CXCL10 is produced by astrocytes upon stimulation of IFN-γ, this feedback loop via astrocytes producing CXCL10 and attracting CXCR3-positive cells is likely critical for maintenance of chronic inflammatory response in HAM/TSP (Sato *et al.*) [133]. In another study by Enose-Akahata *et al*., the B cell-attracting chemokine-1, CXCL13, was found to be increased in CSF of HAM/TSP patients, an increase which correlated with higher HTLV-1-specific antibody responses in CSF and a concomitant decrease of plasma blasts in peripheral blood [134]. Such CSF-associated humoral response could be associated to HAM/TSP progression.

It is known that HTLV-1 modifies the immune response of the host toward other pathogens, increasing susceptibility and worsening clinical manifestations of other infectious diseases. HTLV-1 infection causes an exaggerated production of Th1 cytokines that may down-regulate IL-4, IL-5 and IL-13 production. Treg cells or regulatory molecules may also decrease the host immune response to other pathogens. In addition, impairment of the innate immune response and antigen-presenting cells may contribute to the increased susceptibility to other pathogens seen in HTLV-1 infection. For instance, HTLV-1-transformed ATL cells were unable to boost the production of type I interferons in response to Sendai virus infection. Expression of Tax alone repressed the induction of interferon production by RIG-I + PACT, TBK1 and IRF3. Reciprocal co-immunoprecipitation experiments confirmed the association of Tax with TBK1 kinase that phosphorylates IRF3 [135]. Additionally, Souza *et al.* observed that vaccinated HTLV-1 carriers had a lower antibody production in response to the tetanus toxoid when compared with seronegative controls, suggesting that HTLV-1 is able to decrease antibody production toward a biased antigen [136]. In this study, a decreased expression of co-stimulatory molecules in macrophages from HTLV-1 donors as well as a decreased frequency of dendritic cells in HTLV-1-infected subjects were also observed.

The immunopathogenesis studies presented during the conference clearly testify to the rapid advancement of knowledge concerning the immunologic response in HTLV-1 infection; these studies identify potential biomarkers associated with protection and severe pathology, define molecular mechanisms involved in the downregulation of viral proliferation and suggest novel immunotherapeutic approaches to the treatment of HTLV-1-associated diseases. These studies have been performed with *in vitro* models, animal models and with patient material. Future studies will be designed to translate the basic knowledge into reality for patients who suffer from HTLV-1 infection.

### Molecular and cellular biology

HTLV-1 transmits primarily by cell-to-cell contact. The virus has evolved mechanisms to maximize transmission and escape from the host immune system by clonal proliferation of infected cells. This attribute likely leads to development of ATLL and HAM/TSP. At the stage of transmission of HTLV-1, cell surface glycans play an important role, partly through biofilm-like extracellular viral assembly sites enriched in carbohydrates. By MALDI-TOF MS analysis, Kodama *et al.* demonstrated the increased expression of O-glycans in CD4+ T cells from HAM/TSP patients, which might enhance cell-to-cell transmission of HTLV-1 [137].

Tax is an important viral protein for viral replication and proliferation of infected cells and acts in several ways at transcriptional, post-transcriptional and post-translational levels. Interestingly, data from Barez *et al.* demonstrated that the interaction of Tax with MCM2-7 not only modulates reprogramming of replication origins, but also alters Tax transactivation of the 5’ LTR and thereby viral transcription itself [138]. Tax also inhibits nonsense-mediated mRNA decay (NMD), which might help viral replication (Mocquet *et al*.) [139]. Results from these authors highlight an interaction between Tax and two NMD-associated factors, UPF1 and INT6/EIF3E and additionally underscores Tax-dependent altered morphology of P-bodies and of their content in both NMD factors. In another study by Pène *et al*., analysis of Tax sumoylation allowed the authors to conclude that this post-translational modification was not necessary for Tax-mediated activation of the IKK complex and subsequent NF-κB activation [140].

HBZ is another important player in HTLV-1 pathogenesis. It has been reported that not only the HBZ protein but also HBZ mRNA has important functions (see Barbeau *et al.*, 2013) [141]. HBZ mRNA suppresses apoptosis by inhibiting the induction of p21 and Bax by ultraviolet light (Goicochea *et al*.) [142]. Vernin *et al*. presented another mechanism by which HBZ could act upon cell proliferation and also genetic instability, which involved induced overexpression of oncogenic miR-17 and miR-21 [143]. On the other hand, Gazon *et al.* presented data arguing that HBZ might suppress expression of Dicer 1, and thereby reduce miRNA biogenesis via inhibition of JunD binding to the promoter [144]. Brain-derived neurotrophic factor (BDNF) variant 5 expression is enhanced in HTLV-1-infected cells, via increased transcription caused by the activation domain of HBZ. Expression of the receptor of BDNF, TrkB, is also increased in HTLV-1-infected cells, which leads to activation of a BNDF/TrKB autocrine loop (Polakowski *et al.*) [145]. In addition, HBZ suppresses the canonical Wnt pathway via interaction with TCF-1/LEF-1. However, HBZ enhances Wnt5a expression, which is a ligand for non-canonical Wnt pathway. Thus, HBZ modulates the Wnt pathway towards conditions, which could be favourable for the survival of peripheral T cells (Ma *et al*.) [146].

The long latent period before the onset of ATLL suggests that genetic and epigenetic changes accumulate in the host genome before malignant transformation. In this conference, enhanced expression of the Gem protein (Chevalier *et al.*) [147] and protein methyltransferase 5 (Panfil *et al*.) [148] have both been described in ATL cells. The GEM protein through its ability in altering the cytoskeleton might thus influence cell-to-cell transmission of HTLV-1 and in fact, Chevalier *et al.* have indeed observed GEM-mediated enhancement of conjugates between infected and non-infected cells. The increased level of PRMT5 in ATL cell lines observed by Panfil *et al.* have been linked to cell proliferation and viral replication. In another study, Marçais *et al.* presented data demonstrating that inactivating mutations of TET2 were frequent in patients with ATLL [149].

Post-transcriptional modulation of cellular genes in ATL cells is also of potential interest for the understanding of transformation induced by HTLV-1. Through Exon Chip Human microarrays, Thenoz *et al.* provided evidence for the presence of alternative spliced events (a total of 3642 involved genes) in ATL cell samples differing from cell samples with the untransformed phenotype. These alternatively spliced genes were shown to be part of pathways for p53 signalling, cell cycle and DNA replication [150]. Another post-transcriptional process linked to ATLL has been reported in ATL cells, in which suppressed expression of miR31, which targets the NIK kinase, is linked to activation of NF-κB and increased expression of EZH2 [151]. NF-κB activates EZH2 expression, which leads to suppressed expression of miR31. In addition, EVC1/2 expression is enhanced in ATL cells, which is implicated in activation of the *Hedgehog* signalling pathway (Yamagishi *et al.*) [152].

Studies using the oncogenic virus, BLV, have also helped to identify mechanisms involved in disease mediated by this virus and viral persistence. Durkin *et al*. reported the expression of five viral miRNAs that are implicated in the pathogenesis of BLV-associated disease [153]. In another study, Gillet *et al.* provided an analysis of the clonality of BLV-infected cells after primary infection. Their results revealed massive depletion and selection of BLV-infected cells during the first 2 months [154]. In this early stage, infected cells that have integration sites near a promoter or host gene were eliminated. Nevertheless, clone abundance did correlate with proximity of the provirus to a transcribed region among surviving clones. Based on these observations, integration sites may influence the initial negative selection in the early phase of infection and clone abundance during chronic infection. These effects appear to be similar to those observed in HTLV-1 infection.

### Human Endogenous and emerging Exogenous Retroviruses

In recent decades, we have witnessed the emergence of several simian retroviruses crossing species into humans, some of which, like HIV and HTLV, spread globally and can cause disease. Others, such as simian foamy virus (SFV), and the novel HTLV-3 and −4 viruses discovered in 2006, seem to have a limited spread and an unknown clinical outcome in infected people [155]. Moreover, during the past five years, a mouse retrovirus called XMRV (xenotropic murine leukemia virus-related virus) was reported in prostate cancer and chronic fatigue syndrome, but was shown in subsequent studies to be a laboratory contaminant and not a bona fide human virus [156-158]. In this session, and information presented in several posters, we learned more about the public health importance of these novel emerging human retroviruses and ongoing research to investigate the potential for human endogenous retroviruses to cause disease. New data on the possible contribution of mouse mammary tumor virus (MMTV) to biliary cirrhosis in humans were also presented.

SFV is widely distributed in nonhuman primates (NHPs) and various ape- and monkey-specific strains have been identified in persons exposed to NHPs in various contexts, including occupationally at zoos and research centers, and naturally by hunting, butchering, and keeping NHP pets. Globally, about 138 persons have been identified with SFV infection in a total of 12 countries, including a recent report of two primate workers in China [155,159,160]. Prevalences have ranged from 0.5 – 19%, depending on the severity of the NHP exposure, with a higher prevalence in persons who sustained severe injuries [155]. Nonetheless, little is known about the public health consequences of SFV infection, as only a limited number of close contacts of infected persons have been tested for evidence of person-to-person transmission [155]. Although many SFV-infected persons in the US have reported donating blood before knowing their infection status, an absence of blood-borne transmission was observed in a look back study of leukocyte-reduced blood product recipients from one SFV-infected person in the U.S [155]. All persons identified with SFV infection to date appear healthy [155,160,161].

To investigate the potential for person-to-person transmission, Rua *et al.* determined proviral (DNA) and viral (RNA) loads in saliva and blood specimens (PBMCs and plasma) collected from hunters in Cameroon infected with gorilla SFV (SFVgor) [162]. While low proviral loads were reported in the saliva and PBMCs, viral RNA was not detected in any specimen, suggesting an absence of active viral replication, which may help explain the apparently low transmissibility. Rua *et al.* also investigated the genetic heterogeneity in a subset of the proviral sequences and found that SFV is stable in these individuals, further supporting limited viral replication in these compartments and in infected persons, similar to that reported previously by others [155].

A poster by Filippone *et al.* reported for the first time co-infection of SFVgor-infected hunters in Cameroon, mostly pygmies, with HTLV-1 [109]. Phylogenetic analysis showed that some of the HTLV-1 envelope (*env*) sequences in these individuals were more similar to STLV-1 in monkeys and gorillas, suggesting a recent cross-species transmission from NHPs, similar to that reported in other hunters in Cameroon by Wolfe *et al*. [163]. Any effect that these co-infections will have on the clinical or virologic outcome of each virus in the new host is unknown. A recent study reported an increased SIV-related disease progression in SFV co-infected macaques [164]. In addition, SFV and HIV-1 dual infections have been reported in persons in both Cameroon and the Democratic Republic of Congo (formerly Zaire), which raises the possibility of SFV causing an opportunistic disease in persons immunocompromised by HIV-1 [165]. Thus, a low incidence of disease in SFV-infected persons, like that seen in HTLV infection, cannot be excluded at this point. Additional data and novel study designs are needed to assess both disease associations and transmission risks of SFV in humans.

To date, HTLV-3 has been identified in only four persons living in the forests of Cameroon while HTLV-4 has only been found in a single hunter from Cameroon [163,166-168]. HTLV-4 is the only human retrovirus for which a simian counterpart has not yet been identified. As for SFV, the limited number of infected persons greatly hinders efforts to understand the public health significance of these emerging human retroviruses. However, *in silico, in vitro,* and animal model studies can all help to better understand the pathogenic and transmission potential of these viruses. For example, sequence analysis of the complete genomes of HTLV-3 and HTLV-4 showed that the Tax3 and Tax4 proteins are more similar to those of Tax 1 (HTLV-1) and Tax2 (HTLV-2), respectively, by possessing a PDZ motif in its carboxyl terminus, which is absent in Tax 2 and Tax4 [166,167,169-171]. Since, HTLV-1 is more pathogenic than HTLV-2 and the PDZ domain is essential for the Tax-1 transforming activity, it was suggested that HTLV-3 may have a pathogenic potential similar to that of HTLV-1 [172]. Additional *in vitro* and *in vivo* animal model studies are needed to show that Tax3 does transform infected cells and cause oncogenesis.

Larocque *et al.*[173] reported on the further characterization of the HTLV-3 and −4 antisense proteins (APH3 and APH4) that were initially discovered by *in silico* analysis of their complete genomes and shown *in vitro* by Larocque *et al.* in 2011 to encode proteins that down-regulate Tax-mediated LTR activation, but which have a distinct subcellular localization [174]. Unlike the HTLV-1 HBZ, which has a canonical bZIP domain, APH2 (HTLV-2), APH3 and APH4 have an atypical bZIP-like motif with four leucine heptads followed by a leucine octet instead of the five leucine heptads in HBZ [169]. HBZ and APH2 have been shown to differently affect Jun-dependent transcription and which has also been suggested to explain the differential pathogenic potential of HTLV-1 and HTLV-2. At the meeting, Larocque presented new data that showed, by using co-immunoprecipitation experiments, that APH3 and APH4 interacted with all tested Jun members and that both antisense proteins up-regulated Jun-mediated transactivation of a heterologous promoter. She also showed by mutation analysis that the putative bZIP-like domains and corresponding leucine residues were critical for the Jun factor interaction and modulation of transcription. These studies demonstrated the conservation of the Jun-mediated transcription in HTLV, despite having different cellular and subcellular localizations. More studies are needed to further characterize these novel antisense proteins and to determine their possible role in HTLV-3 and −4 replication and their *in vivo* effect on transmissibility and disease. In addition, these studies may allow a better understanding of the function of HBZ and HTLV-1-associated disease.

ERVs integrated into mammalian germ line genomes many millennia ago [175]. In animals, such as cats (feline leukemia virus), mice (murine leukemia virus and mouse mammary tumor virus), chickens (avian leukosis virus), koalas (koala endogenous retrovirus) and sheep (Jaagsiekte sheep retrovirus), ERVs are associated with malignancies and can grow in human cells *in vitro*[176]. In humans, the association of ERVs (HERVs) with disease has been controversial. About 8% of the human genome is composed of HERVs and, although most are defective, viral expression occurs in human tissues [175,176]. Thus, HERV particles or antibodies have been reported in various autoimmune and neurodegenerative diseases (multiple sclerosis, rheumatoid arthritis, schizophrenia) and cancer (teratocarcinoma, leukemia, ovarian and breast cancer) [175,176]. However, in most reports, it has been difficult to determine if HERV expression causes these diseases or is up-regulated following disease development. Like other retroviruses, HERVs are proposed to possibly affect gene transcription by insertional mutagenesis and recombination, modulation of gene expression via LTR activation, and HERV proteins have been proposed to cause immunosuppression and cell fusion (envelope), autoimmunity (Gag), and control of nuclear factors (Rec and Np9 bind to the promyelocytic leukemia zinc finger to interfere with repression of c-myc) [175,176]. Nonetheless, modern approaches and traditional epidemiological studies are needed to better determine an association of HERV with disease, including microarray and whole transcriptome analyses and testing of coded specimens from rigorously designed case–control studies. One such study was presented in a poster by Babaian and Mager, who have developed a bioinformatic transcriptome data pipeline to assess LTR-based host gene activation using normal and cancer cells [177]. These methods will be useful for evaluating a role of HERV-based LTR activation of host genes in carcinogenesis.

Andrew Mason gave an overview of current data on the role of the human betaretrovirus (HBRV), a virus with high identity to the endogenous betaretrovirus MMTV, in primary biliary cirrhosis (PBC) [178]. In addition to results previously published [179-181], including nested PCR detection of HBRV sequences in persons with PBC and other liver diseases and isolation of virus from PBC patient lymph nodes, phylogenetic analyses of HBRV LTR sequences obtained from liver biopsy specimens of PBC patients were also described. All patient LTR sequences formed three separate clusters with MMTV LTR sequences, suggesting that the inferred topology is inconsistent with a common source of contamination. However, all three clusters had very low bootstrap support, suggesting the inferred genetic relationships could also be random and which is probably a consequence of the short alignment length (62-bp) used for the phylogenetic analysis. Additional data on HBRV integration sites using a nested ligation-mediated PCR technique were presented. HBRV integrated in > 400 sites *in vitro* in Hs578T-infected cells (a human breast cancer cell line) and > 2,700 sites *in vivo* in patient specimens and was integrated on each chromosome. HBRV was reported to preferentially integrate within 100 nucleotides of the NF-κB and SP1 transcription factor motifs. Paradoxically, HBRV integration was also reported in > 10% of negative control specimens and in the Y-chromosome of the infected Hs578T cell line, which is a female cell line and does not have a Y chromosome. More studies are necessary to confirm these findings and to evaluate an association of HBRV or MMTV with human disease. Importantly, as we learned with the de-discovery of XMRV [157,158], mouse DNA can contaminate many reagents and specimens used in these studies and more rigorous study designs, including blinded testing of cases and controls and confirmation of integration performed at independent laboratories, are critically needed to adequately investigate an association of novel human viruses with disease.

Understanding the epidemiology of SFV, HTLV-3 and HTLV-4 and determining whether these retroviruses transmit from person-to-person and cause disease will help to better understand the public health significance of these emerging infections. Testing of close contacts of persons infected with these three retroviruses will help define their transmission potential. While many *in vitro* studies have shown that HTLV-3 resembles HTLV-1 biologically, it remains to be determined if Tax3 can induce cell proliferation either *in vitro* or *in vivo* like Tax1 and thus share the same pathogenic potential. Similarly, more research is needed to further characterize the antisense proteins of all four HTLV groups and to investigate their roles in viral replication, persistence, and pathogenesis. Population-based and expanded molecular epidemiologic studies will determine how widespread these viruses are and determine their natural history in their primate hosts and humans. Specifically, identifying the simian reservoir for STLV-4 will aid in our understanding of the natural history and epidemiology of HTLV-4. More rigorous study designs that include modern technological tools like microarrays and next-generation sequencing will benefit studies to determine associations of HERV and HBRV with disease.

### Virology

Although certain steps in the transmission, persistence and pathogenesis of HTLV-1 and other deltaretroviruses have been well characterized, many aspects of the virology of deltaretroviruses remain poorly understood. For example, few studies published to date have provided insight into the mechanism of viral entry, integration site preference, or the identity of factors that determine whether the virus is expressed or becomes latent. Similarly, little is known about the factors that contribute to the persistence of virally infected cells or to the development of disease in infected individuals. The presentations and posters in the Virology section in this year’s conference provided many novel insights into the biology of deltaretroviruses.

Little is known about the early stage of deltaretroviral infection, including the route of viral entry required for productive infection and the location of proviral integration sites. Jones *et al.* presented evidence that productive infection of dendritic cells by HTLV-1 occurs following entry by macropinocytosis, a type of endocytosis [182]. They observed that reagents that block macropinocytosis dramatically reduce infection of dendritic cells by HTLV-1. In addition, CD4^+^ T cells, which cell-free HTLV-1 does not routinely infect *in vitro*, were productively infected following treatment with a reagent that induces macropinocytosis. Two other studies presented by Sze *et al*. and Alais *et al.* respectively demonstrated that monocytes were not susceptible to HTLV-1 infection due to DNA sensor STING-dependent and SAMHD1-induced apoptosis and that different DC subsets were not equally infected by HTLV-1, possibly accounting for different forms of viral pathogenesis [112,113].

Insight into the integration site preferences of HTLV-1 was provided by Cook *et al*., who used a high-throughput method to characterize proviral integration sites in cells from asymptomatic carriers and individuals with ATLL [183]. They observed that the provirus is typically integrated into transcriptionally active regions of the host genome in individuals with ATLL; this group recently reported that this was true for HTLV-1 integrated in cells from individuals with HAM/TSP [184]. The current study also revealed that not all cases of ATLL are monoclonal. Using this same high-throughput method, Melamed *et al.* performed two studies characterizing HTLV integrated in the genome of PBMC of infected individuals [185,186]. In one study, the group characterized the PVL and clone frequency distribution in CD4^+^ and CD8^+^ T cells in asymptomatic HTLV-1 carriers and individuals with HAM/TSP. HTLV-1-infected CD8^+^ T cells had a clonal distribution distinct from infected CD4^+^ cells: infected CD8^+^ clones were significantly over-represented among the most abundant clones in the blood. In the other study, clone frequency distribution in individuals with HTLV-2, which preferentially infects CD8^+^ T cells, was examined. The clone frequency distribution of HTLV-2 in PBMCs was distinct from that of HTLV-1 and resembled that of HTLV-1-infected CD8^+^ T cells. The authors suggest that, in infections by both viruses, there is a greater degree of selective oligoclonal clonal expansion of infected CD8^+^ T cells. Laydon *et al.* reported that their studies, using a novel mathematical model (DivE) to estimate the number of individual viral clones in the peripheral blood of HTLV-1-infected individuals, indicate that the number is more than two logs higher than previous estimates [187]. Since each unique clone reflects a *de novo* infection event, these observations strongly suggest that there is more cell-to-cell viral spread in infected individuals than previously believed.

Envelope surface (SU) proteins are critical for the initial binding and entry of retroviruses into target cells and are a major target of the immune response. Since retroviral SU proteins are always glycosylated, it is generally believed that the function of this “glycan shield” is to sterically block components of the immune system from interacting with the SU protein. However, a study of the effect of BLV SU glycosylation sites by de Brogniez *et al*. raises the possibility that these modifications may play another role in this protein [188]. They observed that a mutation that blocks glycosylation at residue N230 enhances, rather than inhibits, replication of the virus *in vivo*. Novel insights into the infected lymphocyte populations in BLV-infected, clinically normal cattle were provided by Aida *et al*., who developed a quantitative real-time PCR method that measures the PVL of both known and novel BLV variants in infected animals, like that in HTLV-1-associated ATLL [189]. They observed that PVL correlates with BLV disease progression. Examination of different cell types revealed that, although the cell type believed to be the target of this virus (CD5^+^ IgM^+^ B cells) have the highest PVL, other cell types (CD5^-^ IgM^+^ B cells, CD4^+^ cells, and CD8^+^ T cells) were infected to a greater extent than previously believed.

Another area of active research over the past few years has been the efforts to identify biomarkers that can predict which HTLV-I–infected individuals are at increased risk for developing disease. Two studies presented at this conference by Kagdi *et al.* described the development of an 11-color flow cytometric assay to aid in the diagnosis of, and to monitor, ATLL [33,190]. Using this approach, they discovered that CD4^+^CD25^+^CCR4^+^ T cells can be used as a marker for two aspects of HTLV-1 infection and disease: the frequency of CD4^+^CD25^+^CCR4^+^ T cells correlated with the PVL, and the expression of CD127 on these cells correlated with the ability to achieve remission status.

Previous studies have reported that individuals with HAM/TSP have a higher PVL than asymptomatic individuals, and high PVL has been considered a risk factor for development of HAM/TSP. However, in a long-term study presented by Goncalves *et al.*, PVL only had modest prognostic value in the examined GIPH cohort. Moreover, changes in clinical status and PVL did not coincide in this study: for all five patients for whom PVL was determined both prior to and after development of disease, the median PVL was dramatically higher during the asymptomatic period than after the onset of HAM/TSP [191].

Although current serologic assays are important tools for screening donated blood and identifying individuals infected with HTLV-1 or HTLV-2, these assays have limitations. The development of improved tests is hindered by the lack of a suitable reference panel for these viruses. Morris and Cowan reported on the progress that the World Health Organization has made to develop such a reference panel [192]. Initial screening tests have identified two candidates for HTLV-1a and HTLV-2 for this panel. These are in the process of being formulated into lyophilized preparations, and will soon be distributed to laboratories for testing in a small international collaborative study.

As is the case for cellular genes, the ability of retroviruses to be expressed is based on the state of the chromatin structure. Since the presence of heterochromatin on the integrated HTLV-1 genome blocks transcription of the virus, expression of the virus requires chromatin remodelling. One of the ways that Tax regulates this remodelling involves complexes that require energy from ATP hydrolysis. Using siRNAs, Guendel *et al.* examined the effect of two ATP-chromatin remodelling complexes on transcription [193]. They observed that BAF complexes are regulated by phosphorylation of the Baf 53 subunit, and that PBAF complexes substitute the negative inhibitory BAF complexes needed for activated transcription. Mann *et al.* discovered a novel role for Tax in transcriptional activation involving interaction with an elongation factor [194]. The positive transcription elongation factor b (pTEFb), which is recruited to the LTR by Tax, requires additional proteins for efficient elongation during transcription. Microarray analyses revealed that a single such factor, ELL2, was upregulated by Tax. Further studies revealed that this protein increased Tax-mediated transcription from the HTLV-1 LTR.

Infected cells can silence expression of viruses, including HTLV-1, by microRNAs (miRNAs). It is known that HTLV-1 can dysregulate the cellular RNAi pathway, and at this conference, Van Duyne *et al.* reported that RNAi pathways are altered following interactions of HTLV-1 Tax with Drosha, the enzyme that performs the initial cleavage during processing of miRNAs [195]. They presented evidence that Tax interacts with Drosha, and prevents the initial cleavage of miRNAs by Drosha. They also reported that Drosha was present at lower levels in HTLV-1-infected cell lines and infected primary cells than in uninfected cells.

Studies examining interactions of Tax with host proteins and miRNAs were also presented by Fujikawa *et al.*[196]. Recently, this group reported that the protein EZH2, which is overexpressed in ATL cells, induces constitutive NF-κB activation by repressing a tumor-suppressive miRNA [151]. At this conference, they reported that Tax directly interacts with EZH2 in HTLV-1-infected cells and that independent expression of Tax in primary immune cells results in overexpression of EZH2. Taken together, these results suggest that Tax epigenetically affects gene expression through interaction with EZH2.

Ueno *et al.* presented evidence that a specific polymorphism in the HTLV-1 genome is associated with disease [197]. Individuals infected with the cosmopolitan type A subtype of HTLV-1 in Jamaica and the northern part of Iran are at greater risk for developing HAM/TSP than individuals from Japan infected with this subtype. This group discovered a polymorphism in the pX region of Jamaican and Iranian (J/I) subtype A viruses, which results in an extra 20 amino acids at the C-terminus of Rex. Infectious clones containing the pX region of the J/I subtype A viruses have a higher ratio of full length to doubly-spliced pX mRNA, and produce higher levels of virus, than the Japanese (JP) subtype A virus. These observations suggest that the higher level of HAM/TSP in individuals from Jamaica and Iran may reflect higher viral loads in these individuals due to a decreased ability of the mutant Rex protein in the J/I subtype A viruses to control expression of the virus.

In many infected individuals, HTLV-1 is transcriptionally silent, and the majority of leukemic cells from ATLL patients do not express the *tax* gene transcript. Studies by Cook *et al.* examined the factors contributing to the silencing of *tax* expression in cells from individuals with ATLL [183]. They observed that Tax was not expressed in 65% of the cases of ATLL examined, and that this lack of expression was the result of promoter deletion, *tax* gene nonsense mutations, or *tax* promoter hypermethylation.

Cells secrete endosome-derived microvesicles called exosomes, and recent studies have shown that some viruses use exosomes to enhance their spread. Narayanan *et al.* reported that, in addition to proteins usually associated with exosomes, exosomes derived from HTLV-1-infected cells contain gp46 and Tax, as well as inflammatory mediators including IL-6 and IL-10 [198]. Treatment of naïve cells with exosomes secreted from HTLV-1-infected cells induced a response in reactive oxygen species production. The authors suggest that these exosomes may play a role in pathogenesis of HTLV-1 infection.

The likelihood of long-term progression to AIDS in individuals co-infected with HTLV-2 and HIV-1 is significantly lower than in individuals infected with HIV-1 alone. Barrios *et al.* found that expression of Tax-2 in HIV-1-infected PBMCs resulted in a significant reduction in HIV-1 produced by the cells, and that this decreased expression was preceded by increased levels of CC-chemokines [199]. The authors state that these observations, along with a similar but less robust inhibition of HIV-1 infection by Tax-1, suggest that Tax plays a role in generating antiviral responses against HIV-1.

NF-κB signaling plays a pivotal role in Tax-1-mediated transformation and ATLL leukemogenesis. Huleihel and Shvarzbeyn observed that the natural product Propolis (PE) and its active component, caffeic acid phenethyl ester, inhibit the activation of NF-κB-dependent promoters by Tax-1, and that PE could also efficiently inhibit the activation of SRF- and CREB-dependent promoters [200]. Xiang *et al.* found that treatment of HTLV-1-transformed T cells with the anti-helminthic molecule niclosamide induced degradation of Tax with subsequent suppression of transcription of HTLV-1 viral genes and apoptosis [201].

## Conclusions

At the 16^th^ International Conference on Human Retrovirology: HTLV and Related Viruses, researchers presented their exciting and original findings, which will have an important impact on both clinical and fundamental research. Scientists and clinicians had the opportunity to actively engage with patient representatives from Brazil, UK and Japan, who openly shared their concerns and needs with the scientific community at the opening ceremony. Following the formation of the Clinical Trial Groups during the previous meeting held in Leuven, Belgium, in 2011, a specific clinical trial workshop was organised, allowing clinicians and researchers to specifically discuss ongoing and future drug trials. We are looking forward to the 17^th^ International Conference on Human Retrovirology: HTLV and Related Viruses, which will be held in Les Trois-Ilets, Martinique in June 2015.

## Abbreviations

AC: Asymptomatic control; ADCC: Antibody-dependent cell-mediated cytotoxicity; ATLL: Adult T-cell leukemia/lymphoma; AZT: Zidovudine; BLV: Bovine leukemia virus; CTL: Control; CR: Complete remission; CNS: Central nervous system; CSF: Cerebral spinal fluid; GZMA: Granzyme A; HAM/TSP: HTLV-1-associated myelopathy/ tropical spastic paraparesis, HBRV, Human betaretrovirus; HDAC: Histone desacetylase; HERV: Human endogenous retroviruses; HIV: Human immunodeficiency virus; HTLV: Human T-cell leukemia virus; IFN: Interferon; IS: Integration site; MDDC: Monocyte-derived dendritic cell; MMTV: Mouse mammary tumor virus; NHP: Non-human primate; NMD: Nonsense-mediated decay; PBMC: Peripheral blood mononuclear cell; PVL: Proviral load; SFV: Simian foamy virus; SIV: Simian immunodeficiency virus; STLV: Simian T-cell leukemia virus; TSDR: Treg-specific methylation region; VPA: Valproate; XMRV: Xenotropic murine leukemia virus-related virus.

## Competing interest

None of the authors declare any conflict of interests. Use of trade names is for identification only and does not imply endorsement by the U.S. Department of Health and Human Services, the Public Health Service, or the Centers for Disease Control and Prevention. The findings and conclusions in this report are those of the authors and do not necessarily represent the views of the Centers for Disease Control and Prevention.

## Author’s contribution

F.M. and A.B. wrote the Clinical Trial Session Groups section; T.W. wrote the Clinical Research section; L.R. wrote the Animal Models section; E.M. wrote the Epidemiology section; J.H. and E.C. wrote the Immunology section; M.M. wrote the Molecular and Cellular Biology section; W.M.S. wrote the Human Endogenous and Emerging Exogenous Retroviruses section; K.J. wrote the Virology Section. B.B. edited the manuscript. All authors read and approved the final manuscript.
